# Dynamic activation of bone morphogenetic protein signaling in collagen-induced arthritis supports their role in joint homeostasis and disease

**DOI:** 10.1186/ar2518

**Published:** 2008-09-24

**Authors:** Melina Daans, Rik JU Lories, Frank P Luyten

**Affiliations:** 1 Laboratory for Skeletal Development and Joint Disorders, Division of Rheumatology, Department of Musculoskeletal Sciences, Katholieke Universiteit Leuven, Herestraat 49 box 813, Leuven 3000, Belgium

## Abstract

**Introduction:**

Rheumatoid arthritis is a chronic systemic autoimmune disease affecting peripheral joints and leading to loss of joint function. The severity and outcome of disease are dependent on the balance between inflammatory/destructive and homeostatic or repair pathways. Increasing evidence suggests a role for bone morphogenetic protein (BMP) signaling in joint homeostasis and disease.

**Methods:**

Activation of BMP signaling in collagen-induced arthritis as a model of rheumatoid arthritis was studied by immunohistochemistry and Western blot for phosphorylated SMAD1/5 at different time points. Expression of different BMP ligands and noggin, a BMP antagonist, was determined on synovium and cartilage extracts of arthritic knees, at different time points, with quantitative polymerase chain reaction. At the protein level, BMP2 and BMP7 were studied with immunohistochemistry. Finally, the effect of anti-tumor necrosis factor-alpha (TNFα) treatment on the expression of BMP2, BMP7, and growth and differentiation factor-5 (GDF5) in synovium and cartilage of arthritic knees was investigated.

**Results:**

A time-dependent activation of the BMP signaling pathway in collagen-induced arthritis was demonstrated with a dynamic and characteristic expression pattern of different BMP subfamily members in synovium and cartilage of arthritic knees. As severity increases, the activation of BMP signaling becomes more prominent in the invasive pannus tissue. BMP2 is present in cartilage and the hyperplastic lining layer. BMP7 is found in the sublining zone and inflammatory infiltrate. Treatment with etanercept slowed down progression of disease, but no change in expression of GDF5, BMP2, and BMP7 in synovium was found; in the cartilage, however, blocking of TNFα increased the expression of BMP7.

**Conclusions:**

BMP signaling is dynamically activated in collagen-induced arthritis and is partly TNFα-independent. TNFα blocking increased the expression of BMP7 in the articular cartilage, possibly enhancing anabolic mechanisms. Different types of source and target cells are recognized. These data further support a role for BMP signaling in arthritis.

## Introduction

Rheumatoid arthritis (RA) is a chronic and systemic autoimmune disease that affects mainly the peripheral joints. Synovitis with infiltration of inflammatory cells, synoviocyte proliferation, and accelerated angiogenesis triggers the formation of destructive pannus tissue and osteoclast activation that lead to erosion of cartilage and bone with progressive loss of joint function [1]. From a molecular point of view, the severity and prognosis of RA are dependent on the balance between inflammatory or destructive pathways and homeostatic or repair pathways [2,3]. Molecular signaling pathways, essential for tissue development and growth, such as bone morphogenetic proteins (BMPs), are likely to play a role in tissue homeostasis and repair [4]. However, inappropriate or exaggerated activation of such pathways may also lead to pathology [5-8].

BMPs are members of the transforming growth factor-beta superfamily, a group of structurally related growth and differentiation factors. Their pleiotropic effects on different cell types steer many pre- and postnatal processes, such as cell differentiation, proliferation, adhesion, motility, and apoptosis [9-11]. BMPs were originally discovered as proteins that ectopically induce cartilage and bone formation *in vivo *[12] and are important during the embryonic development of articular joints [13-16]. Almost 30 BMPs are described and classified into several subgroups according to their structural similarities [17]. Binding of a dimeric BMP ligand to type I and type II BMP receptors typically activates a downstream signaling cascade involving either SMAD family member (SMAD) molecules or mitogen-activated protein kinases. In the classical and most extensively studied pathway, the receptor-ligand complex will phosphorylate the intracellular receptor-SMAD1 and -SMAD5 molecules. These will form a complex with common SMAD4, which translocates to the nucleus, binds to DNA, and directs the transcription of BMP target genes. BMP signaling is regulated at different levels: by ligand diversity, by secreted extracellular BMP antagonists, by inhibitory SMADs, and by nuclear corepressors and coactivators [18,19].

Different BMPs have been demonstrated in the synovium of RA patients [20-22] but their function and their target cells are not yet clear. BMPs have a chondroprotective role in different animal models of RA [23]. In the present study, we investigated the activation of BMP signaling and expression patterns of different BMP ligands and antagonists in collagen-induced arthritis (CIA). CIA is a well-established mouse model of RA, which develops in susceptible mouse strains following immunization with heterologeous type II collagen (CII) emulsified in complete Freund's adjuvant (CFA) and shares both immunological and pathological features with human RA [24]. Our data highlight the relevance of BMP signaling in the joint and provide a basis for further studies on the role of specific BMPs in RA.

## Materials and methods

### Animal studies

Eight-week-old male DBA/1J mice were purchased from Janvier Laboratories (Le Genest-Saint-Isle, France). All experiments were approved by the Ethics Committee for Animal Research (Katholieke Universiteit Leuven, Leuven, Belgium). For induction of arthritis, chicken sternal cartilage CII (Sigma-Aldrich, Bornem, Belgium) was dissolved at 2 mg/mL in phosphate-buffered saline (PBS)/0.1 M acetic acid, stirred overnight (O/N) at 6°C, and emulsified with an equal volume of CFA (1 mg/mL) (Sigma-Aldrich). One hundred microliters of the emulsion (0.1 mg of CII) was injected intradermally at the base of the tail. At day 21 after immunization, mice received an intraperitoneal booster injection of 100 μL of CII (2 mg/mL). At day 25, the onset of arthritis was synchronized by an intraperitoneal injection of 100 μL of lipopolysaccharide (500 μg/mL in PBS) (Sigma-Aldrich) [25]. Mice were sacrificed at different time points (day 0, 20, 27, 33, 40, and 47 after immunization) for immunohistochemistry and protein and RNA expression assays. In additional experiments, mice were injected daily with 100 μL of soluble tumor necrosis factor-alpha (TNFα) receptor etanercept/PBS (250 μg/mL) (Wyeth Pharmaceuticals, Louvain-la-Neuve, Belgium) intraperitoneally (or PBS alone as negative control) from day 29 onwards. The severity score was determined daily according to the scoring system of Backlund and colleagues [26]. Mice were sacrificed at day 35.

### RNA extraction, cDNA synthesis, and quantitative polymerase chain reaction analysis of synovium and cartilage samples

At each time point and at the end of the TNFα blocking experiment, synovium and cartilage samples were dissected, separated, and used for RNA extraction. RNA was isolated using a Nucleospin RNAII kit (Macherey-Nagel, Düren, Germany) and reverse-transcribed using a Revert-Aid H Minus First strand cDNA synthesis kit (Fermentas, St. Leon-Rot, Germany) according to the manufacturers' instructions. For quantitative analysis, real-time polymerase chain reaction (PCR) was performed in duplicate using the Rotor-gene 6000 detection system (Corbett Research, Westburg, Leusden, The Netherlands). Gene expression of mouse *BMP2*, *BMP4*, *BMP6*, *BMP7*, growth and differentiation factor-5(*GDF5*), Noggin (*NOG*), and *TNFα *were studied using assay-on-demand primer/probe sets (Applied Biosystems, Lennik, Belgium). Expression was normalized to mouse housekeeping gene *GAPDH *(glyceraldehydes-3-phosphate dehydrogenase) using the comparative threshold method [27]. In kinetic experiments, data were further normalized to baseline levels.

### Protein extraction and Western blot analysis of whole knees

At each time point, three sets of three pooled knees were used for protein extraction. Whole knees were weighed and homogenized (CAT homogenizer X120; CAT Ingenieurbüro M. Zipperer GmbH, Staufen, Germany) in 1 mL of cell extraction buffer (Biosource Europe, Nivelles, Belgium) supplemented with 5% Proteinase Inhibitor Cocktail (Sigma-Aldrich) and 1 mM PMSF (phenylmethylsulfonyl fluoride) (Sigma-Aldrich). Protein extracts were normalized to wet weight in an appropriate volume of cell extraction buffer. Samples were analyzed under reduced conditions (0.1 M DTT [1,4-dithiothreitol]). Samples were boiled for 5 minutes at 95°C, chilled on ice, and loaded onto a 4% to 12% Bis-Tris gel (Invitrogen Corporation, Carlsbad, CA, USA). Electrophoresis was carried out into a commercially available running buffer (NuPage MES SDS Running buffer; Invitrogen Corporation) at 130 V for 10 minutes in the beginning, followed by 25 minutes at 200 V. Proteins were transferred on a prewet PVDF (polyvinylidene difluoride) membrane (Millipore S.A./N.V., Brussels, Belgium) for 70 minutes at 30 V in a transfer buffer containing 0.4 M glycine, 0.5 M Tris base, 0.01 M SDS, and 200 mL/L methanol. Nonspecific binding sites were blocked in Tris-buffered saline/0.1% Tween (TBST) (wash buffer) with 5% milkpowder (BlottoA) (Santa Cruz Biotechnology, Inc., Santa Cruz, CA, USA) for 1 hour at room temperature (RT). Blots were then probed O/N at 4°C with polyclonal antibody against phosphorylated SMAD1/5 (P-SMAD1/5) or SMAD5 (Cell Signaling Technology, Inc., Danvers, MA, USA) (1:1,000 in TBST/5% bovine serum albumin [BSA]) and thereafter incubated with an appropriate horseradish peroxidase (HRP)-conjugated secondary antibody (Jackson ImmunoResearch Laboratories, Inc., West Grove, PA, USA) at a dilution of 1:5,000 in TBST/5% milkpowder for 1 hour at RT. For detection, a chemiluminescent substrate (Western Lightning; PerkinElmer Life and Analytical Sciences, Inc., Waltham, MA, USA) was applied on the membrane. Blots were visualized using an LAS-3000 mini CCD (charge-coupled device) camera using an exposure time of 15 minutes. Densitometry measurements were done using digital image densitometry analysis (ImageJ; National Institutes of Health, Bethesda, MD, USA). As positive controls, mouse mesenchymal progenitor C2C12 cells were stimulated with recombinant BMP2 (300 ng/mL for 30 minutes). As negative controls, blots were incubated with secondary antibody alone (data not shown).

### Immunohistochemistry

Knees and ankles were dissected, formaldehyde/PBS-fixed O/N, decalcified with Decal (3 days at RT) (Serva, Heidelberg, Germany) or EDTA (ethylenediaminetetraacetic acid) (0.5 M in PBS, pH 7.5) (10 changes at 4°C), and embedded in paraffin. For immunohistochemistry, paraffin sections (5 μm thick) were deparaffinized with Histo-Clear (National Diagnostics, Atlanta, GA, USA) and methanol. For antigen retrieval, sections were incubated 2 hours at RT in 0.1 M sodium citrate/0.1 M citric acid. Endogenous peroxidase was quenched by incubating the slides for 10 minutes in 3% H_2_O_2 _in water (BMP7 and P-SMAD1/5) or 3% H_2_O_2 _in methanol (BMP2). Sections were washed three times in PBS/0.1% Triton (BMP7 and P-SMAD1/5) or in TBST (wash buffer) (BMP2) and blocked 30 minutes at RT in 20% donkey serum (BMP7, BMP2) or 20% goat serum (P-SMAD1/5) in wash buffer and were incubated O/N at 4°C with primary antibody at a final concentration of 10 μg/mL chicken anti-BMP7 (Pfizer Inc, New York, NY, USA), 2 μg/mL goat anti-BMP2 (Santa Cruz Biotechnology, Inc.), 1:50 dilution of rabbit anti-P-SMAD1/5 (Cell Signaling Technology, Inc.) or with isotype control (chicken, goat, and rabbit IgG) (Santa Cruz Biotechnology, Inc.) or serum (Dako, Glostrup, Denmark) at an appropriate concentration in wash buffer. Sections were then washed three times with wash buffer and incubated for 30 minutes at RT with secondary antibody. For BMP7 immunostaining, the secondary antibody was an HRP-conjugated anti-chicken (Jackson ImmunoResearch Europe Ltd, Newmarket, Suffolk, UK) diluted 1:100. For BMP2 immunostaining, a biotinylated donkey anti-goat at 1:400 dilution was used, followed by streptavidin anti-HRP (LSAB kit) (Dako) (30 minutes at RT). For P-SMAD1/5 immunostaining, an ABC kit (Vecta stain rabbit IgG [Vector laboratories Ltd., Peterborough, UK]) was used for signal amplification. Liquid DAB (3,3'-diaminobenzidine) substrate chromogen system (Dako) was used as a peroxidase substrate. Sections were counterstained with hematoxylin. Adjacent sections were stained with hematoxylin and eosin (H&E). GDF5 immunohistochemistry was not performed on these samples as we found that different commercially available antibodies showed a lack of specificity.

### Statistical analysis

Where appropriate (n > 3), results were analyzed with SPSS 15.0 (SPSS Inc., Chicago, IL, USA) with the unpaired non-parametric Mann-Whitney *U *test. Statistically significant differences were defined as *P *values of less than 0.05.

## Results

### Activation of bone morphogenetic protein signaling in collagen-induced arthritis

**Figure 1:**
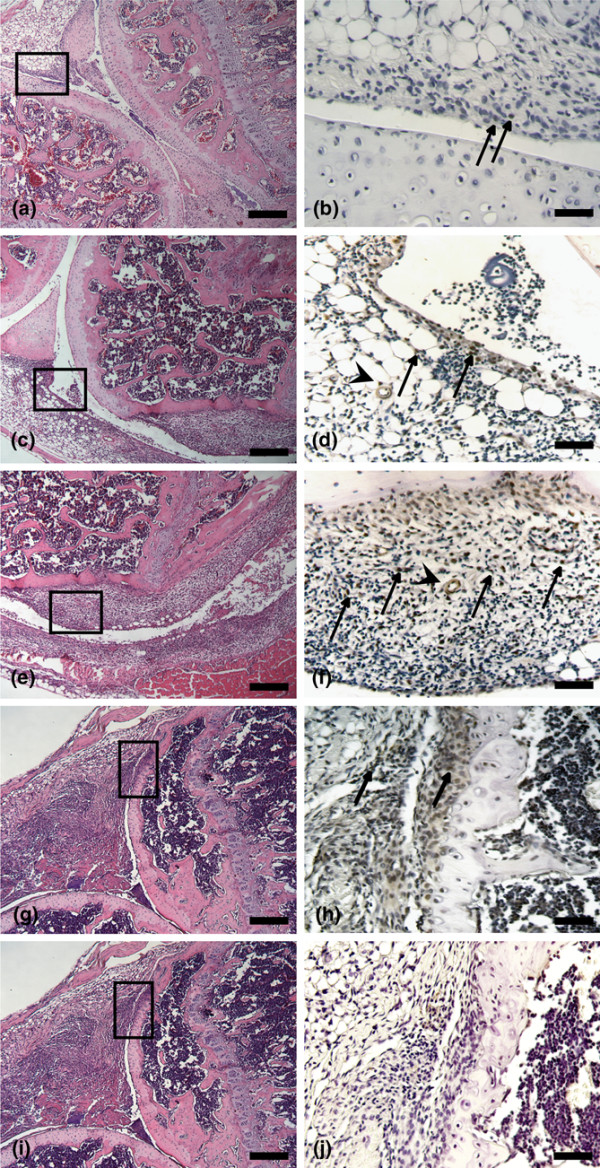
Activation of bone morphogenetic protein signaling in the mouse knee during collagen-induced arthritis. Immunohistochemistry for P-SMAD1/5 in the initial phase **(a, b)**, with increasing severity **(c-f)**, and in the destructive phase of collagen-induced arthritis **(g, h) **and IgG control **(j) **is shown. Scoring was done on sections stained with hematoxylin and eosin (a, c, e, g, i). Boxes indicate areas that are magnified in P-SMAD1/5 stainings (b, d, f, h). Arrows indicate P-SMAD1/5-positive cells, and arrowheads indicate P-SMAD1/5-positive blood vessels. Bars = 200 μm (a, c, e, g, i), 50 μm (d, f, h), and 25 μm (b). P-SMAD, phosphorylated Smad family members; SMAD, Smad family members.

Activation of BMP signaling during the course of CIA was visualized at different time points by immunohistochemistry for P-SMAD1/5 (Figure 1b,d,f,h). Histo-morphological scoring of adjacent H&E-stained sections was used to assess the severity of disease (Figure 1a,c,e,g,i). In the initial phase of CIA (exsudate score 1 and infiltration and pannus formation score 0), only a few P-SMAD1/5-positive cells were visible, mostly in the lining layer of the synovium (Figure 1b). With increasing severity, characterized by infiltration of inflammatory cells and synovial hyperplasia (exsudate score 1, infiltration score 3, and pannus formation score 1), P-SMAD1/5-positive cells became apparent in the subintimal zone of the synovium. Also, a few blood vessels and articular chondrocytes showed nuclear P-SMAD1/5 staining (Figure 1d–f). Eventually, in the destructive phase (pathology exsudate score 2, infiltration score 3, and pannus formation score 2), P-SMAD1/5-positive cells were identified in the invading pannus tissue (Figure 1h). In contrast, healthy knees showed almost no P-SMAD1/5-positive cells (data not shown) and IgG controls were negative (Figure 1j).

**Figure 2:**
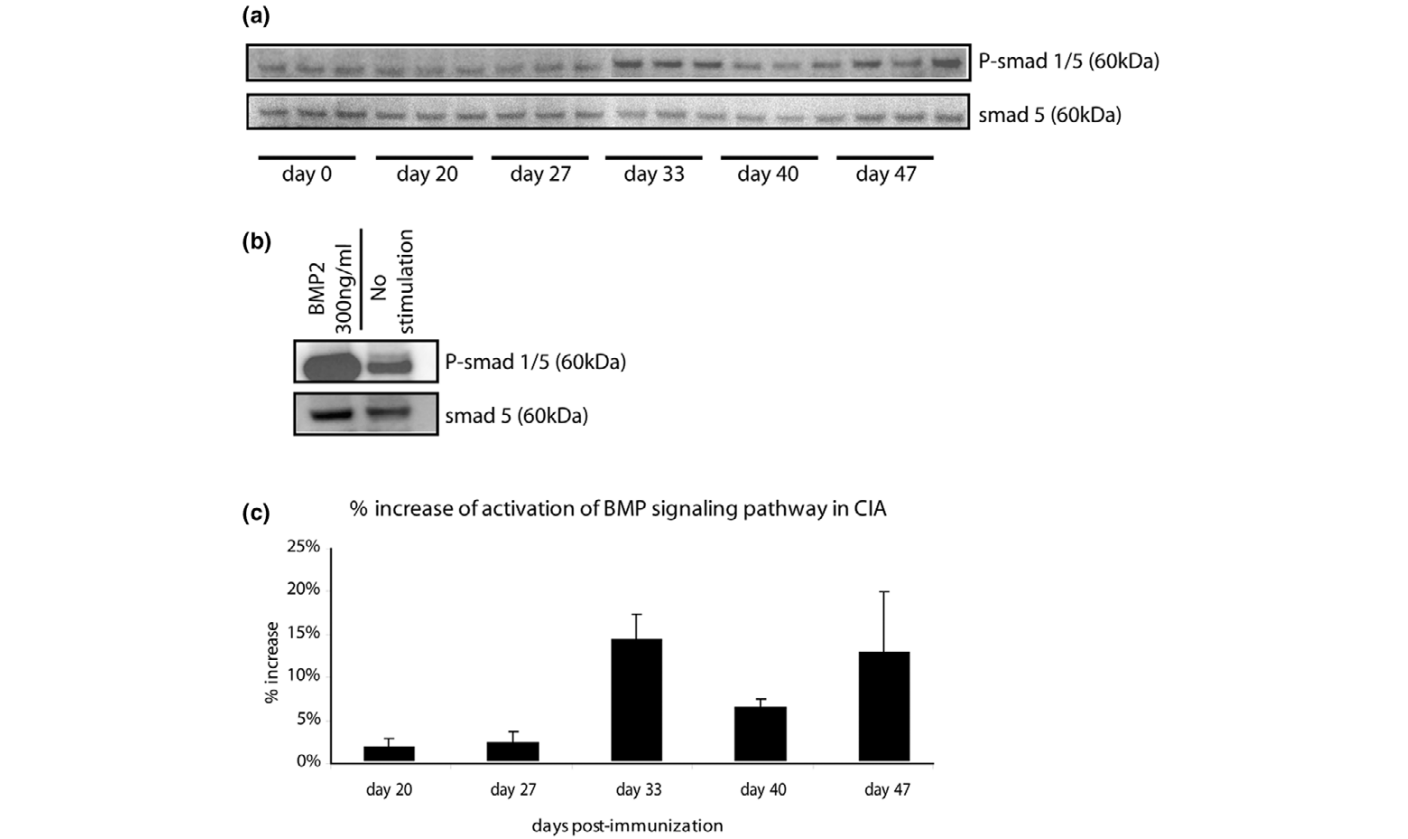
Quantification of P-SMAD1/5 expression during collagen-induced arthritis (CIA) by Western blot. **(a) **Western blot for P-SMAD1/5 and SMAD5 on mouse whole knee extracts at different time points in CIA, normalized to wet weight (n = 3 sets of pooled knees per time point). **(b) **Positive control of Western blot for P-SMAD1/5 and SMAD5 on C2C12 cells unstimulated or stimulated with bone morphogenetic protein-2 (BMP2) (300 ng/mL) for 30 minutes. Ten micrograms of protein was loaded. **(c) **Analysis of the activation of the BMP signaling pathway, which was measured as the inverted density of P-SMAD1/5 bands (normalized to density of SMAD5 bands) (n = 3 sets of 3 pooled knees per time point). Data are presented as mean ± standard deviation. P-SMAD, phosphorylated Smad family members; SMAD, Smad family members.

Activation of BMP signaling in CIA was further semi-quantified by Western blot on pooled protein extracts from whole knees at different time points (day 0, 20, 27, 33, 40, and 47 of the experiment). Positive controls were obtained by stimulating C2C12 cells with recombinant BMP2 (300 ng/mL) for 30 minutes. Mouse knee extracts (three per time point, each pooled from three different knees) were normalized to wet weight. A specific 60-kDa band was found in all samples (Figure 2a). Negative controls showed no band of this size (data not shown). C2C12 cells stimulated with BMP2 had a higher expression of P-SMAD1/5 than unstimulated cells (Figure 2b). We further studied the relative amount of phosphorylated and therefore activated SMAD1/5 as compared with total SMAD5 levels. Densities of P-SMAD1/5 and SMAD5 bands of mouse samples were quantified using digital image analysis densitometry software. (ImageJ, National Institutes of Health, Bethesda, MD, USA) Normalized density of P-SMAD1/5 bands per time point was consistently upregulated (three samples versus three samples) from day 33 onwards as compared with baseline (Figure 2c). These semi-quantitative Western blot data thus corroborated our immunohistochemical results, supporting more active BMP signaling in the joint as CIA progresses.

### Kinetics of bone morphogenetic protein ligands in collagen-induced arthritis – mRNA level

**Figure 3:**
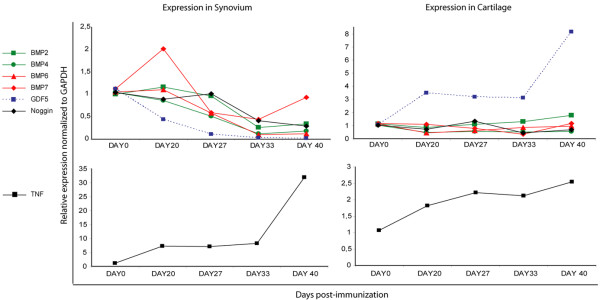
Quantitative polymerase chain reaction of different bone morphogenetic protein (BMP) ligands in synovium and cartilage during collagen-induced arthritis. Relative expression of BMP subfamily members *BMP2 *and *BMP4 *(green), *BMP6 *and *BMP7 *(red), *GDF5 *(dashed blue), and noggin (black) (upper panel) and *TNFα *(lower panel) at different time points in collagen-induced arthritis synovium (left) and cartilage (right) towards day 0, no immunisation. Expression was normalized to *GAPDH*. (n = 3 sets of 2 pooled mice per time point.) Data are presented as mean relative expression. GAPDH, glyceraldehydes-3-phosphate dehydrogenase; GDF5, growth and differentiation factor-5; TNFα, tumor necrosis factor-alpha.

Quantitative PCR for different BMP ligands and BMP antagonist *NOG *in both synovium and cartilage extracts (three sets per time point, extracts from two mice pooled per set) showed a dynamic expression pattern with upregulation or downregulation of these molecules during the course of CIA (day 0, 20, 27, 33, and 40) as compared with healthy controls (Figure 3). As expected, expression of *TNFα *in the synovium gradually increased during the course of the disease process. *BMP7 *was upregulated with peak expression around day 20. The expression levels of *BMP2*, *BMP4*, *BMP6*, and *NOG *were stable around day 20 and decreased gradually, in contrast to *GDF5 *expression, which was downregulated abruptly around day 20. As gene expression levels are normalized to a housekeeping gene, they are also likely to be influenced by the increased number of cells in the synovium. In the articular cartilage, an increase in *TNFα *was visualized, though to a milder extent, but we found a striking increase in *GDF5 *expression, whereas the other molecules showed a more or less stable expression pattern. At day 40, *BMP2 *was very mildly upregulated in the articular cartilage. Again, due to normalization, expression levels are likely to be influenced by the decreased number of cells in the cartilage, due to chondrocyte cell death.

### Kinetics of bone morphogenetic protein ligands in collagen-induced arthritis – protein level

**Figure 4:**
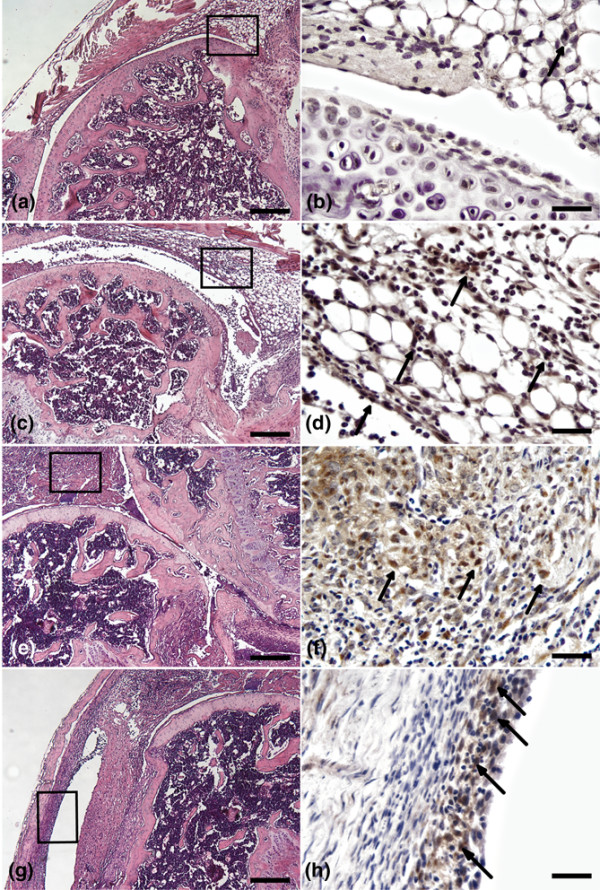
Presence of bone morphogenetic protein-2 (BMP2) during collagen-induced arthritis. Immunohistochemistry in the initial phase **(a, b)**, with increasing severity **(c-f)**, and in the destructive phase of collagen-induced arthritis **(g, h) **is shown. Severity scoring was done on sections stained with hematoxylin and eosin. Boxes indicate areas that are magnified in BMP2 stainings. Arrows indicate BMP2-positive cells in synovium. Bars = 200 μm (a, c, e, g) and 25 μm (b, d, f, h).

**Figure 5:**
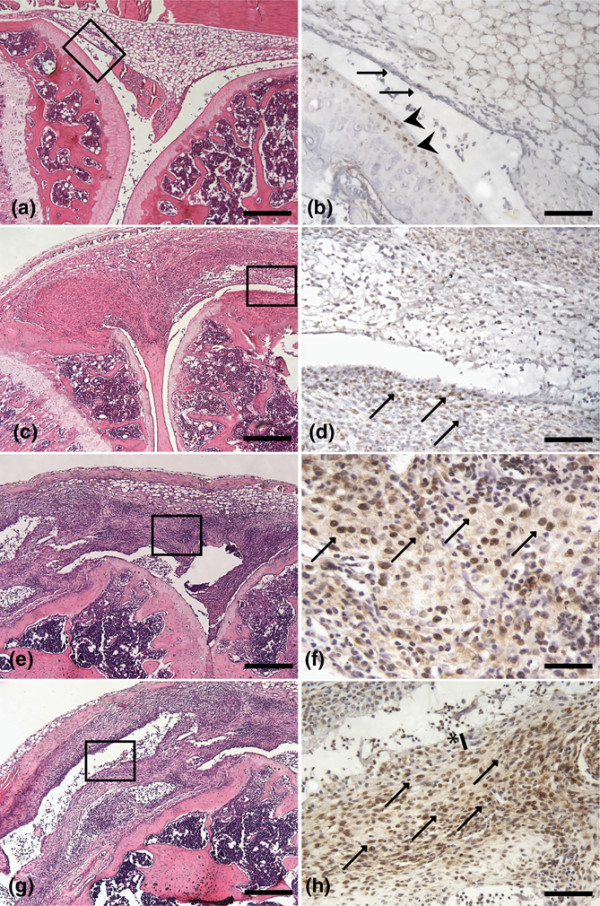
Presence of bone morphogenetic protein-7 (BMP7) during collagen-induced arthritis. Immunohistochemistry in the initial phase **(a, b)**, with increasing severity **(c-f)**, and in the destructive phase of collagen-induced arthritis **(g, h**) is shown. Severity scoring was done on sections stained with hematoxylin and eosin. Boxes indicate areas that are magnified in BMP7 stainings. Arrows indicate BMP7-positive cells in synovium, and arrowheads indicate BMP7-positive cells in articular cartilage. Asterisk indicates gradual clearing of the synovial lining layer. Bars = 200 μm (a, c, e, g), 50 μm (b, d), and 25 μm (f).

The gene expression data prompted us to further study BMP2 and BMP7 protein levels in the arthritic joint. These prototype BMPs belong to different subfamilies preferentially binding to distinct type I and type II receptor complexes. Immunohistochemical analysis of affected mouse knees revealed BMP2 expression predominantly in the hyperplastic lining layer of synovium and in the articular cartilage (Figure 4b,h). BMP2 was less expressed but still present in some cells in the subintimal zone (Figure 4d,f). The BMP7 staining pattern in mouse knee joint was influenced by disease severity. Mild inflammation (Figure 5a) was associated with discrete BMP7 staining in the lining layer of synovium (Figure 5b) and in superficial chondrocytes in the articular cartilage (Figure 5b). With increasing severity (Figure 5c,e), BMP7 positivity became more pronounced in the lining layer and subintima (Figure 5d,f). Eventually, in the destructive phase (Figure 5g), BMP7 positivity appeared more and more in the subintima, away from the lining layer (Figure 5h).

### Effect of tumor necrosis factor-alpha blocking on bone morphogenetic protein expression levels in collagen-induced arthritis

**Figure 6:**
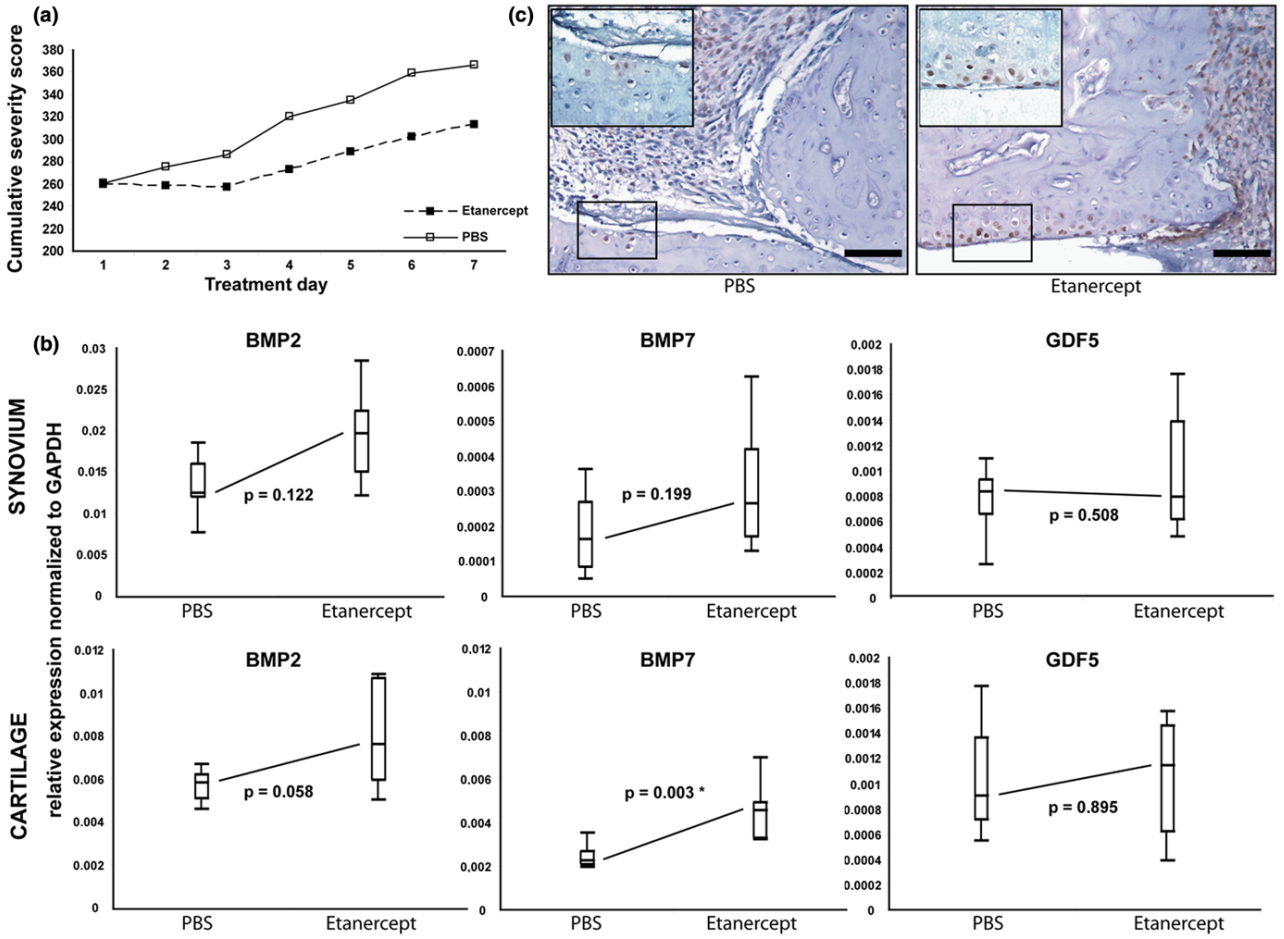
Effect of tumor necrosis factor-alpha blocking on bone morphogenetic protein (BMP) expression levels in collagen-induced arthritis. **(a) **Cumulative severity scores of mice treated with phosphate-buffered saline (PBS) (solid line) or etanercept (dashed line) (n = 9 animals per group). Data are shown as total group scores at each time point. **(b) **Quantitative polymerase chain reaction of *BMP2 *(left), *BMP7 *(middle), and *GDF5 *(right) on synovium (upper) and cartilage (lower) of mice treated with PBS or etanercept (n = 9 animals per group). Relative expression is normalized to *GAPDH*. Data are presented as median, quartile, and percentile (Mann-Whitney *U *test *P < 0.05). **(c) **Expression of BMP7 on the protein level in articular cartilage from mice treated with PBS or etanercept. Immunohistochemistry for BMP7 on ankle sections. Insets represent higher-magnification views (3:1) of the boxed areas. Bar = 50 μm. GAPDH, glyceraldehydes-3-phosphate dehydrogenase; GDF5, growth and differentiation factor-5.

As BMP expression was recently reported in the human TNF transgenic mouse model, we further investigated the effect of blocking TNFα on expression of BMPs in synovium and cartilage, using daily intraperitoneal injections of etanercept or PBS (as control) during clinically apparent CIA (n = 9 mice per group). Six-day treatment reduced severity of arthritis, as shown by the cumulative severity scores (Figure 6a). At endpoint, synovium and cartilage were collected and mRNA expression levels of *BMP2*, *BMP7*, and *GDF5 *were compared between the two groups. In the synovium, expression levels of *BMP2*, *BMP7*, and *GDF5 *were not significantly changed. In cartilage, however, etanercept treatment raised the expression levels of *BMP7 *significantly (*P *= 0.003). *BMP2 *and *GDF5 *expression was not affected (Figure 6b). Immunohistochemistry confirmed the increased levels of BMP7 in the cartilage (Figure 6c).

## Discussion

In the present study, we demonstrated a dynamic activation of the BMP signaling pathway, as detected by P-SMADs, in a mouse model of RA, CIA. The activation pattern is dependent on the stage of disease, starting, in the initial phase, at the synovial lining layer, gradually shifting toward the subintimal region and eventually, in the destructive phase, persisting in pannus tissue. Moreover, similar dynamic expression levels were shown for different BMP ligands in CIA. A more extensive study of BMP2 and BMP7, different BMP subfamily members, revealed for both BMPs a distinct and dynamic pattern. In contrast to BMP2, which is restricted mainly to the synovial lining layer and articular cartilage, BMP7 resembles the P-SMAD1/5 positivity pattern very closely (Figure 1). Upon TNF blockade, the expression of BMP7 was increased in the articular cartilage of affected joints, whereas in the synovium the expression levels of BMP2, BMP7, and GDF5 were unchanged, suggesting that at least part of the regulation of BMP expression is TNF-independent. This suggests that, although some BMPs are upregulated under inflammatory conditions, other autocrine and paracrine mechanisms may be important and may sustain BMP expression during the arthritic disease process [3,28]. In addition, the upregulation of BMP7 seen after anti-TNF treatment, which has an inhibitory effect on cartilage and bone destruction, supports an anabolic effect of TNF blockage. Until now, this association was shown with the Wnt pathway in arthritic mice, in which inhibition of TNF decreased the expression of Dickkopf, a Wnt antagonist, known for its neutralizing effect on anabolic mechanisms while supporting catabolic pathways of joint destruction [29], and with melanoma inhibitory activity in RA patients, a chondrocyte-specific molecule with anabolic characteristics, which has a decreased expression under pro-inflammatory cytokine conditions [30].

Results of earlier studies on BMP expression in RA already speculated on a potential role for BMPs in RA. Our group showed an increased expression of BMP2 and BMP6 in the synovium of RA patients and illustrated their association with apoptosis of synoviocytes [20]. BMP4 and BMP5, however, are reduced in the synovium of RA patients as compared with healthy patients [21]. BMP7 has been demonstrated in the synovial fluid of RA patients and levels are correlated with severity of disease [31]. Marinova-Mutafchieva and colleagues [22] observed BMP type Ia (activin-like receptor kinase-3) receptor-positive mesenchymal cells in the synovium of RA patients, and recently we described different BMP target cells, including mostly fibroblast-like synoviocytes and the vascular-perivascular niche, in synovial biopsies of RA patients [28]. An effective treatment of arthritis resulted in an overall reduction of active BMP signaling. However, the pathway remained active and the relative number of P-SMAD1/5-positive cells did not change, suggesting that indeed part of BMP regulation is inflammation-independent.

Animal models of arthritis are increasingly used to address the role of BMPs in disease pathogenesis. Our group previously studied the role of BMP signaling in joint homeostasis and repair by modulating the BMP signaling pathway in different mouse models of chronic arthritis [23]. *NOG *haploinsufficiency provided protection for articular cartilage against destruction in methylated BSA-induced arthritis and delayed the progression from cartilage to bone formation in a mouse model of spontaneous ankylosing enthesitis [23] by enhancing BMP signaling. Blaney Davidson and colleagues [32] showed that BMP2 is associated with cartilage protection, chondrogenesis, and osteophyte formation in an animal model of osteoarthritis, and Badlani and colleagues [33] demonstrated that BMP7 protected the articular cartilage in a rabbit model of osteoarthritis, confirming the *in vitro *pro-anabolic and anti-catabolic properties of BMP7 as proposed by Chubinskaya and colleagues [34] and Fan and colleagues [35]. Overexpression of *NOG *rendered the cartilage more vulnerable in two mouse models of destructive arthritis (methylated BSA and CIA) [23] and inhibited the onset and progression of remodeling arthritis [8].

In contrast, in these overexpression or genetic models, we have not seen detectable differences in synovitis [23]. Recently, Bobacz and colleagues [36] demonstrated a differential expression of GDF5 and BMP7 in articular cartilage and synovium of h*TNF*tg mice. They found an increased expression of BMP7 and GDF5 in the synovium of h*TNF*tg mice along with a decrease of both genes in articular cartilage. Based on their *in vitro *data, they concluded that a decrease in the cartilage could compromise cartilage repair while an increase of BMP7 and GDF5 in the synovium might contribute to synovial hypertrophy. However, Steenvoorden and colleagues [37] showed that transforming growth factor-beta induced an epithelial-mesenchymal transition-like phenomenon, which apparently precedes synovial hypertrophy, and which can be inhibited *in vitro *by adding BMP7. Contrasting data also exist on the function of BMP7 in other disease models. In inflammatory bowel disease [38] and acute renal failure [39], BMP7 treatment reduces the severity of the pathogenesis and favors healing. BMP7 inhibits tumor growth in some forms of cancer [40]. In contrast, BMP7 can promote cell invasion and tumor growth [41] and directs cancer to metastasis [42,43] or exerts malignant fibrinogenic effects [44].

## Conclusion

The data presented reveal that BMP signaling is activated during the course of CIA, following a specific pattern, and may be partly independent of TNFα. Furthermore, TNF blocking possibly enhances repair mechanisms via upregulation of BMP7. Our data also confirm that different cells in the synovium and cartilage are a target for BMP signaling. Taken together, the current data suggest a chondroprotective effect of BMPs on articular cartilage, but the biological effect of different BMPs in distinct synovial compartments may be more complex and further functional studies are warranted.

## Abbreviations

BMP: bone morphogenetic protein; BSA: bovine serum albumin; CFA: complete Freund's adjuvant; CIA: collagen-induced arthritis; CII: collagen type II; GDF5: growth and differentiation factor-5; H&E: hematoxylin and eosin; HRP: horseradish peroxidase; NOG: noggin; O/N: overnight; PBS: phosphate-buffered saline; PCR: polymerase chain reaction; P-SMAD: phosphorylated Smad family members; RA: rheumatoid arthritis; RT: room temperature; SMAD: Smad family members; TBST: Tris-buffered saline/0.1% Tween; TNFα: tumor necrosis factor-alpha.

## Competing interests

The authors declare that they have no competing interests.

## Authors' contributions

MD performed and analyzed the experiments. RJUL and FPL participated in the design and coordination of the study, helped to draft the manuscript, and gave their final approval of the version to be published. All authors read and approved the final manuscript.

## Authors' information

MD is the recipient of a fellowship from the Institute for the Promotion of Innovation through Science and Technology in Flanders (IWT Vlaanderen). RJUL is the recipient of a postdoctoral fellowship from the Research Foundation Flanders.

## References

[B1] Firestein GS (2003). Evolving concepts of rheumatoid arthritis. Nature.

[B2] Feldmann M, Brennan FM, Maini RN (1996). Rheumatoid arthritis. Cell.

[B3] Luyten FP, Lories RJ, Verschueren P, de Vlam K, Westhovens R (2006). Contemporary concepts of inflammation, damage and repair in rheumatic diseases. Best Pract Res Clin Rheumatol.

[B4] Lories RJ, Luyten FP (2005). Bone morphogenetic protein signaling in joint homeostasis and disease. Cytokine Growth Factor Rev.

[B5] Sancho E, Batlle E, Clevers H (2004). Signaling pathways in intestinal development and cancer. Annu Rev Cell Dev Biol.

[B6] Hsu MY, Rovinsky S, Penmatcha S, Herlyn M, Muirhead D (2005). Bone morphogenetic proteins in melanoma: angel or devil?. Cancer Metastasis Rev.

[B7] Yoshikawa H, Nakase T, Myoui A, Ueda T (2004). Bone morphogenetic proteins in bone tumors. J Orthop Sci.

[B8] Lories RJ, Derese I, Luyten FP (2005). Modulation of bone morphogenetic protein signaling inhibits the onset and progression of ankylosing enthesitis. J Clin Invest.

[B9] Hogan BL (1996). Bone morphogenetic proteins in development. Curr Opin Genet Dev.

[B10] Ferguson CM, Miclau T, Hu D, Alpern E, Helms JA (1998). Common molecular pathways in skeletal morphogenesis and repair. Ann N Y Acad Sci.

[B11] Liu Z, Luyten FP, Lammens J, Dequeker J (1999). Molecular signaling in bone fracture healing and distraction osteogenesis. Histol Histopathol.

[B12] Urist MR (1965). Bone: formation by autoinduction. Science.

[B13] Thomas JT, Kilpatrick MW, Lin K, Erlacher L, Lembessis P, Costa T, Tsipouras P, Luyten FP (1997). Disruption of human limb morphogenesis by a dominant negative mutation in CDMP1. Nat Genet.

[B14] Luyten FP (1997). Cartilage-derived morphogenetic protein-1. Int J Biochem Cell Biol.

[B15] Polinkovsky A, Robin NH, Thomas JT, Irons M, Lynn A, Goodman FR, Reardon W, Kant SG, Brunner HG, Burgt I van der, Chitayat D, McGaughran J, Donnai D, Luyten FP, Warman ML (1997). Mutations in CDMP1 cause autosomal dominant brachydactyly type C. Nat Genet.

[B16] Thomas JT, Lin K, Nandedkar M, Camargo M, Cervenka J, Luyten FP (1996). A human chondrodysplasia due to a mutation in a TGF-beta superfamily member. Nat Genet.

[B17] Ducy P, Karsenty G (2000). The family of bone morphogenetic proteins. Kidney Int.

[B18] Massague J, Chen YG (2000). Controlling TGF-beta signaling. Genes Dev.

[B19] Balemans W, Van Hul W (2002). Extracellular regulation of BMP signaling in vertebrates: a cocktail of modulators. Dev Biol.

[B20] Lories RJ, Derese I, Ceuppens JL, Luyten FP (2003). Bone morphogenetic proteins 2 and 6, expressed in arthritic synovium, are regulated by proinflammatory cytokines and differentially modulate fibroblast-like synoviocyte apoptosis. Arthritis Rheum.

[B21] Bramlage CP, Haupl T, Kaps C, Ungethum U, Krenn V, Pruss A, Muller GA, Strutz F, Burmester GR (2006). Decrease in expression of bone morphogenetic proteins 4 and 5 in synovial tissue of patients with osteoarthritis and rheumatoid arthritis. Arthritis Res Ther.

[B22] Marinova-Mutafchieva L, Taylor P, Funa K, Maini RN, Zvaifler NJ (2000). Mesenchymal cells expressing bone morphogenetic protein receptors are present in the rheumatoid arthritis joint. Arthritis Rheum.

[B23] Lories RJ, Daans M, Derese I, Matthys P, Kasran A, Tylzanowski P, Ceuppens JL, Luyten FP (2006). Noggin haploinsufficiency differentially affects tissue responses in destructive and remodeling arthritis. Arthritis Rheum.

[B24] Courtenay JS, Dallman MJ, Dayan AD, Martin A, Mosedale B (1980). Immunisation against heterologous type II collagen induces arthritis in mice. Nature.

[B25] Terato K, Harper DS, Griffiths MM, Hasty DL, Ye XJ, Cremer MA, Seyer JM (1995). Collagen-induced arthritis in mice: synergistic effect of *E. coli *lipopolysaccharide bypasses epitope specificity in the induction of arthritis with monoclonal antibodies to type II collagen. Autoimmunity.

[B26] Backlund J, Nandakumar KS, Bockermann R, Mori L, Holmdahl R (2003). Genetic control of tolerance to type II collagen and development of arthritis in an autologous collagen-induced arthritis model. J Immunol.

[B27] Giulietti A, Overbergh L, Valckx D, Decallonne B, Bouillon R, Mathieu C (2001). An overview of real-time quantitative PCR: applications to quantify cytokine gene expression. Methods.

[B28] Verschueren PC, Lories RJ, Daans M, Theate I, Durez P, Westhovens R, Luyten FP Detection, identification and *in vivo *treatment responsiveness of BMP activated cell populations in the synovium of rheumatoid arthritis patients. Ann Rheum Dis.

[B29] Diarra D, Stolina M, Polzer K, Zwerina J, Ominsky MS, Dwyer D, Korb A, Smolen J, Hoffmann M, Scheinecker C, Heide D van der, Landewe R, Lacey D, Richards WG, Schett G (2007). Dickkopf-1 is a master regulator of joint remodeling. Nat Med.

[B30] Vandooren B, Cantaert T, van Lierop MJ, Bos E, De Rycke L, Veys EM, De Keyser F, Bresnihan B, Luyten FP, Verdonk PC, Tak PP, Boots AH, Baeten D Melanoma Inhibitory Activity, a biomarker related to chondrocyte anabolism, is reversibly suppressed by proinflammatory cytokines in rheumatoid arthritis. Ann Rheum Dis.

[B31] Chubinskaya S, Frank BS, Michalska M, Kumar B, Merrihew CA, Thonar EJ, Lenz ME, Otten L, Rueger DC, Block JA (2006). Osteogenic protein 1 in synovial fluid from patients with rheumatoid arthritis or osteoarthritis: relationship with disease and levels of hyaluronan and antigenic keratan sulfate. Arthritis Res Ther.

[B32] Blaney Davidson EN, Vitters EL, Kraan PM van der, Berg WB van den (2006). Expression of transforming growth factor-beta (TGFbeta) and the TGFbeta signalling molecule SMAD-2P in spontaneous and instability-induced osteoarthritis: role in cartilage degradation, chondrogenesis and osteophyte formation. Ann Rheum Dis.

[B33] Badlani N, Inoue A, Healey R, Coutts R, Amiel D (2008). The protective effect of OP-1 on articular cartilage in the development of osteoarthritis. Osteoarthritis Cartilage.

[B34] Chubinskaya S, Kawakami M, Rappoport L, Matsumoto T, Migita N, Rueger DC (2007). Anti-catabolic effect of OP-1 in chronically compressed intervertebral discs. J Orthop Res.

[B35] Fan Z, Chubinskaya S, Rueger DC, Bau B, Haag J, Aigner T (2004). Regulation of anabolic and catabolic gene expression in normal and osteoarthritic adult human articular chondrocytes by osteogenic protein-1. Clin Exp Rheumatol.

[B36] Bobacz K, Sunk IG, Hayer S, Amoyo L, Tohidast-Akrad M, Kollias G, Smolen JS, Schett G (2007). Differentially regulated expression of growth differentiation factor 5 and bone morphogenetic protein 7 in articular cartilage and synovium in murine chronic arthritis: Potential importance for cartilage breakdown and synovial hypertrophy. Arthritis Rheum.

[B37] Steenvoorden MM, Tolboom TC, Pluijm G van der, Löwik C, Visser CP, DeGroot J, Gittenberger-DeGroot AC, DeRuiter MC, Wisse BJ, Huizinga TW, Toes RE (2006). Transition of healthy to diseased synovial tissue in rheumatoid arthritis is associated with gain of mesenchymal/fibrotic characteristics. Arthritis Res Ther.

[B38] Maric I, Poljak L, Zoricic S, Bobinac D, Bosukonda D, Sampath KT, Vukicevic S (2003). Bone morphogenetic protein-7 reduces the severity of colon tissue damage and accelerates the healing of inflammatory bowel disease in rats. J Cell Physiol.

[B39] Vukicevic S, Basic V, Rogic D, Basic N, Shih MS, Shepard A, Jin D, Dattatreyamurty B, Jones W, Dorai H, Ryan S, Griffiths D, Maliakal J, Jelic M, Pastorcic M, Stavljenic A, Sampath TK (1998). Osteogenic protein-1 (bone morphogenetic protein-7) reduces severity of injury after ischemic acute renal failure in rat. J Clin Invest.

[B40] Notting I, Buijs J, Mintardjo R, Horst G van der, Vukicevic S, Lowik C, Schalij-Delfos N, Keunen J, Pluijm G van der (2007). Bone morphogenetic protein 7 inhibits tumor growth of human uveal melanoma *in vivo*. Invest Ophthalmol Vis Sci.

[B41] Rothhammer T, Poser I, Soncin F, Bataille F, Moser M, Bosserhoff AK (2005). Bone morphogenic proteins are overexpressed in malignant melanoma and promote cell invasion and migration. Cancer Res.

[B42] Alarmo EL, Korhonen T, Kuukasjarvi T, Huhtala H, Holli K, Kallioniemi A (2007). Bone morphogenetic protein 7 expression associates with bone metastasis in breast carcinomas. Ann Oncol.

[B43] Buijs JT, Henriquez NV, van Overveld PG, Horst G van der, Que I, Schwaninger R, Rentsch C, Ten Dijke P, Cleton-Jansen AM, Driouch K, Lidereau R, Bachelier R, Vukicevic S, Clézardin P, Papapoulos SE, Cecchini MG, Löwik CW, Pluijm G van der (2007). Bone morphogenetic protein 7 in the development and treatment of bone metastases from breast cancer. Cancer Res.

[B44] Tacke F, Gabele E, Bataille F, Schwabe RF, Hellerbrand C, Klebl F, Straub RH, Luedde T, Manns MP, Trautwein C, Brenner DA, Scholmerich J, Schnabl B (2007). Bone morphogenetic protein 7 is elevated in patients with chronic liver disease and exerts fibrogenic effects on human hepatic stellate cells. Dig Dis Sci.

